# Parvalbumin interneuron loss mediates repeated anesthesia-induced memory deficits in mice

**DOI:** 10.1172/JCI159344

**Published:** 2023-01-17

**Authors:** Patricia Soriano Roque, Carolina Thörn Perez, Mehdi Hooshmandi, Calvin Wong, Mohammad Javad Eslamizade, Shilan Heshmati, Nicole Brown, Vijendra Sharma, Kevin C. Lister, Vanessa Magalie Goyon, Laura Neagu-Lund, Cathy Shen, Nicolas Daccache, Hiroaki Sato, Tamaki Sato, Jeffrey S. Mogil, Karim Nader, Christos G. Gkogkas, Mihaela D. Iordanova, Masha Prager-Khoutorsky, Heidi M. McBride, Jean-Claude Lacaille, Linda Wykes, Thomas Schricker, Arkady Khoutorsky

**Affiliations:** 1 Department of Anesthesia and; 2 School of Human Nutrition, McGill University, Montreal, Canada.; 3 Department of Neurosciences, Center for Interdisciplinary Research on Brain and Learning (CIRCA) and Research Group on Neural Signaling and Circuitry (GRSNC), Université de Montréal, Montreal, Canada.; 4 Department of Biochemistry, McGill University, Montreal, Canada.; 5 Medical Nanotechnology and Tissue Engineering Research Center, Shahid Beheshti University of Medical Sciences, Tehran, Iran.; 6 Montreal Neurological Institute,; 7 Department of Psychology, Faculty of Science, and; 8 Alan Edwards Centre for Research on Pain, McGill University, Montreal, Canada.; 9 Biomedical Research Institute, Foundation for Research and Technology–Hellas, University Campus, Ioannina, Greece.; 10 Department of Psychology/Centre for Studies in Behavioural Neurobiology, Concordia University, Montreal, Canada.; 11 Department of Physiology and; 12 Faculty of Dental Medicine and Oral Health Sciences, McGill University, Montreal, Canada.

**Keywords:** Neuroscience, Apoptosis, Memory

## Abstract

Repeated or prolonged, but not short-term, general anesthesia during the early postnatal period causes long-lasting impairments in memory formation in various species. The mechanisms underlying long-lasting impairment in cognitive function are poorly understood. Here, we show that repeated general anesthesia in postnatal mice induces preferential apoptosis and subsequent loss of parvalbumin-positive inhibitory interneurons in the hippocampus. Each parvalbumin interneuron controls the activity of multiple pyramidal excitatory neurons, thereby regulating neuronal circuits and memory consolidation. Preventing the loss of parvalbumin neurons by deleting a proapoptotic protein, mitochondrial anchored protein ligase (MAPL), selectively in parvalbumin neurons rescued anesthesia-induced deficits in pyramidal cell inhibition and hippocampus-dependent long-term memory. Conversely, partial depletion of parvalbumin neurons in neonates was sufficient to engender long-lasting memory impairment. Thus, loss of parvalbumin interneurons in postnatal mice following repeated general anesthesia critically contributes to memory deficits in adulthood.

## Graphical abstract

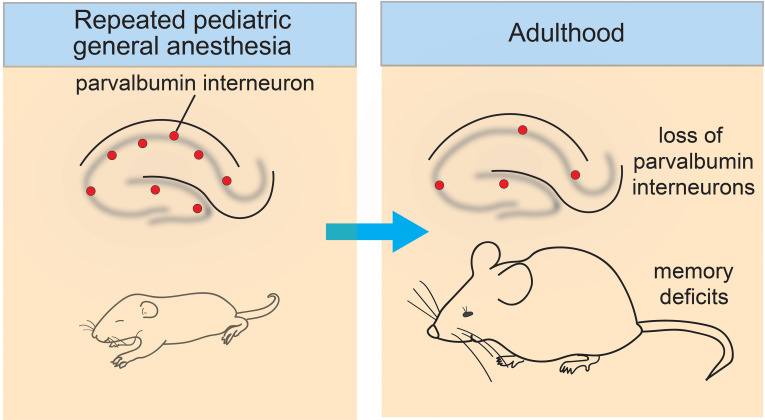

## Introduction

Studies in rodents (1–4) and nonhuman primates (5, 6) have shown that prolonged or repeated, but not short-term (7–9), general anesthesia in juvenile animals leads to long-lasting memory impairment (1, 4–6). Furthermore, pediatric epidemiological studies have linked repeated or prolonged general anesthesia to cognitive and behavioral abnormalities, including learning disabilities and attention deficit/hyperactivity disorder (10–13). The accumulating evidence in animal models as well as epidemiological reports in the pediatric population have raised clinical concerns regarding the use of prolonged or repeated general anesthesia in young children, prompting the US FDA to issue a safety warning on its use in children less than 3 years of age (14). In the US, 14.9% of children undergo general anesthesia at least once before age 3, and of those, 26% are exposed to repeated or prolonged anesthesia (15). Mechanistically, repeated anesthesia-induced apoptosis, which occurs in less than 2% of neurons in the brain of juvenile animals (1), was proposed to contribute to persistent memory deficits (16). However, it remains unclear how the loss of such a small population of neurons following repeated anesthesia can cause cognitive deficits that persist into adulthood and whether a specific subset of neurons preferentially undergoes apoptosis and subsequent cell death. Here, we show that repeated general anesthesia in postnatal mice induces preferential apoptosis and partial loss of parvalbumin-positive (Pvalb-positive) interneurons in the hippocampus. Blocking the loss of Pvalb interneurons by ablating a proapoptotic protein, mitochondrial anchored protein ligase (MAPL), in this cell type prevented repeated anesthesia-induced deficits in hippocampal inhibition and long-term memory formation in adulthood. Conversely, ablation of Pvalb neurons caused long-lasting memory deficits. Thus, our data demonstrate that loss of Pvalb interneurons mediates repeated general anesthesia–induced memory deficits in adulthood.

## Results

### Preferential apoptosis of Pvalb neurons.

**Figure 1:**
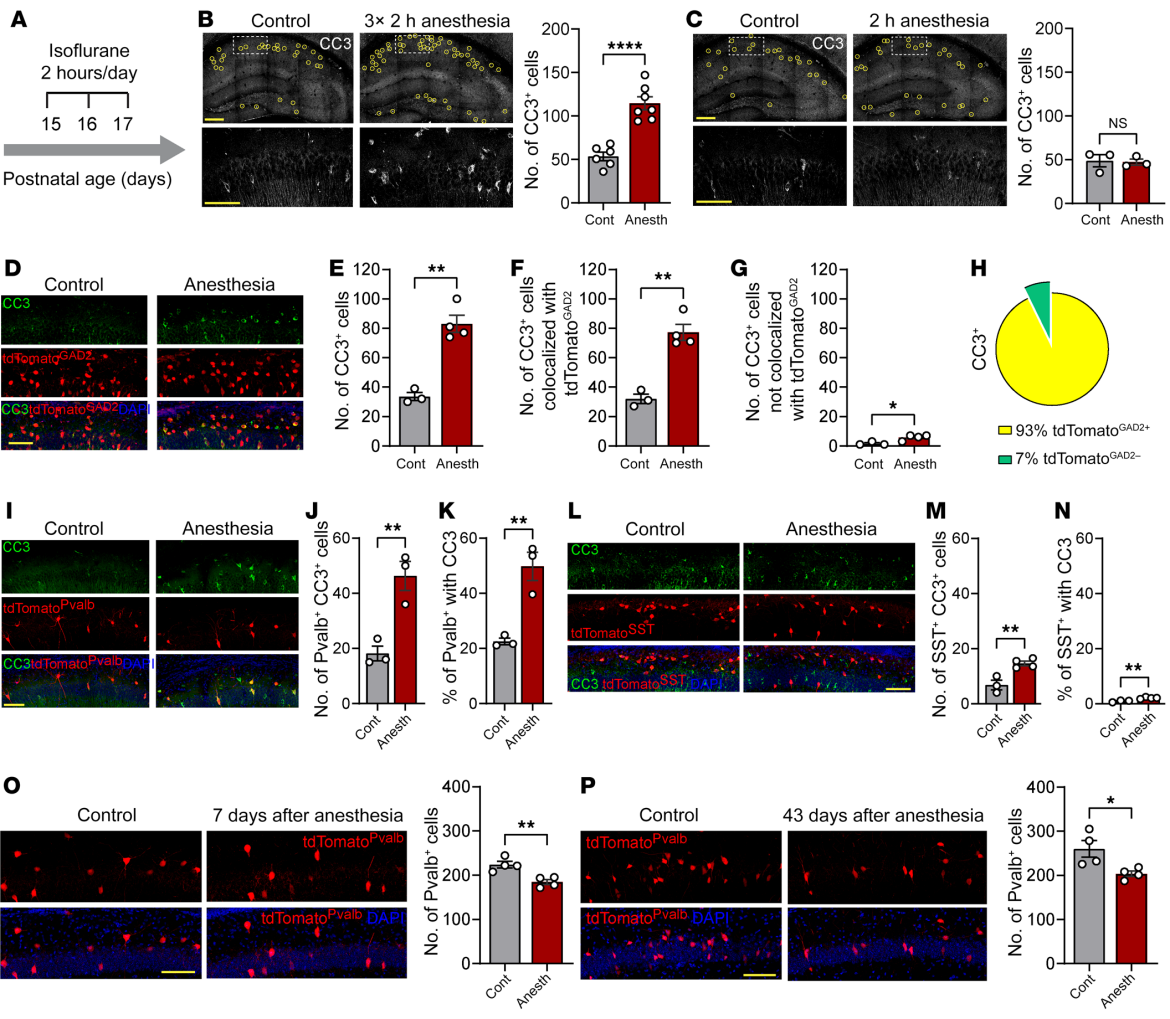
Pvalb-positive interneurons undergo preferential apoptosis and cell death after repeated general anesthesia in juveniles. (**A**) Experimental design. (**B**) Immunostaining for the apoptotic marker CC3 reveals that repeated anesthesia in juvenile mice increases the number of CC3^+^ cells in the hippocampus. Brains were PFA fixed 1 hour after the last anesthesia session on P17. Each circle marks a CC3^+^ cell. Bottom images show higher magnification of the areas marked by rectangles in the top low-magnification images. Scale bars: 200 μm (top); 100 μm (bottom). (Right) Quantification of CC3^+^ cells in the hippocampus. Each data point represents an individual male animal. Anesth, anesthesia. (**C**) Single 2-hour exposure to isoflurane anesthesia. (**D**) Repeated anesthesia preferentially increases CC3 in GAD2^+^ neurons in tdTomato^GAD2^ male mice. Shown are the total number of hippocampal CC3^+^ neurons (**E**). (**F**) Number of neurons colocalized with tdTomato^GAD2^. (**G**) Number of neurons showing no colocalization with tdTomato^GAD2^. (**H**) 93% of all CC3^+^ hippocampal neurons are inhibitory (GAD2^+^) neurons. (**I**) Repeated anesthesia induces CC3 upregulation in a significant fraction of Pvalb neurons in tdTomato^Pvalb^ male mice. (**J**) Total number of hippocampal Pvalb CC3^+^ neurons. (**K**) Percentage of Pvalb neurons with CC3. (**L**) Repeated anesthesia induces CC3 upregulation in SST neurons in tdTomato^SST^ male mice. (**M**) Total number of hippocampal SST CC3^+^ neurons. (**N**) Percentage of SST neurons with CC3. (**O** and **P**) Reduced number of Pvalb neurons in the hippocampus of tdTomato^Pvalb^ male mice at day 7 (**O**) and day 43 (**P**) after repeated anesthesia. Scale bars: 100 μm (**B**–**P**). Statistics are based on unpaired 2-tailed *t* test in all panels. Each data point represents an individual male animal. Data are represented as mean ± SEM. *P < 0.05; **P < 0.01; ****P < 0.0001.

To reveal which neuronal cell types undergo apoptosis following repeated anesthesia in early postnatal mice, we assessed apoptosis using a well-established apoptotic marker, cleaved caspase-3 (CC3) (17). We used reporter mice expressing tdTomato in inhibitory neurons under the GAD2 promoter (tdTomato^GAD2^) to distinguish between excitatory and inhibitory neurons. The tdTomato^GAD2^ mice were subjected to 2 hours of isoflurane anesthesia per day for 3 consecutive days, for a total of 6 hours, starting at P15, Figure 1A). This anesthesia protocol was selected based on previous studies (18, 19) so that models would mimic children with substantial cumulative exposure to anesthesia. In accordance with previous reports (2, 20), monitoring oxygen saturation (sPO_2_%), respiratory rate (RR), and heart rate (HR) showed stable hemodynamic status of animals during anesthesia at this age (Supplemental Figure 1, A–C; supplemental material available online with this article; https://doi.org/10.1172/JCI159344DS1). Consistent with previous studies, we found that apoptosis in the dorsal hippocampus, the area implicated in spatial memory formation (21), was induced after 3 consecutive days of anesthesia exposure (P17; Figure 1B), whereas no increase in the number of apoptotic cells was observed after a single 2-hour anesthesia session (P15; Figure 1C). Intriguingly, 93% of CC3-positive cells in the repeated anesthesia-exposed mice were inhibitory neurons (Figure 1, D–H). Three major subclasses of inhibitory interneurons are Pvalb, somatostatin (SST), and vasoactive intestinal polypeptide (VIP) positive (22). To reliably detect these neuronal subclasses in the brain, even in apoptotic cells when the expression of cell identity markers may change, we generated reporter mice expressing tdTomato in each subpopulation (tdTomato^Pvalb^, tdTomato^SST^, and tdTomato^VIP^) and subjected them to repeated anesthesia (2 hours of isoflurane anesthesia per day for 3 consecutive days) at P15–P17. Apoptosis was significantly induced in Pvalb (Figure 1, I–K) and SST (Figure 1, L–N) neurons. No CC3 immunoreactivity was detected in VIP neurons in control or anesthesia-exposed mice (Supplemental Figure 1D and Supplemental Figure 2, G–I). Normalization to the total number of cells in each subclass showed that anesthesia-induced CC3 immunoreactivity was detected in a significantly higher proportion of Pvalb neurons (~50% of all Pvalb neurons; Figure 1K) than SST neurons (~2% of all SST neurons, Figure 1N). An increased number of CC3^+^ Pvalb neurons after anesthesia was detected in all hippocampal areas except the subiculum (Supplemental Figure 2, A–C), whereas apoptosis induction in SST neurons was restricted to the dentate gyrus (Supplemental Figure 2, D–F). Repeated anesthesia also induced an increase in the number of CC3-positive cells in the cortex (Supplemental Figure 2J), with substantial apoptosis in Pvalb neurons (Supplemental Figure 2, K and L).

**Figure 2:**
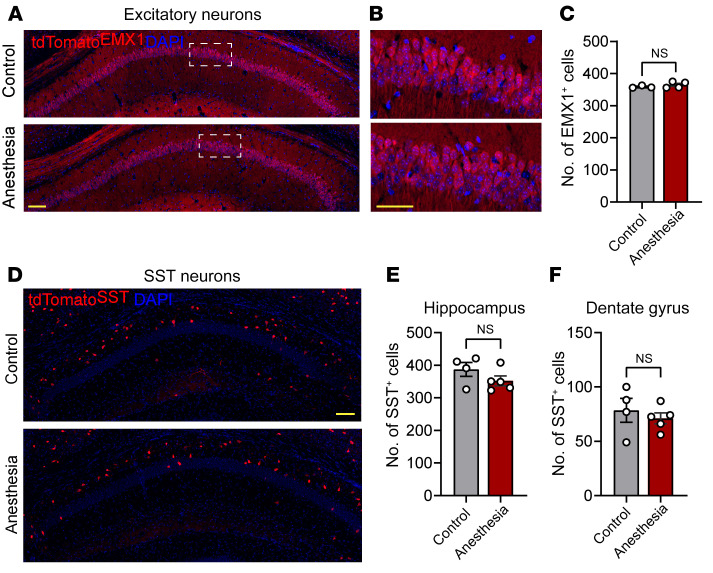
No change in the number of excitatory and SST neurons after repeated general anesthesia. Male mice expressing tdTomato in excitatory neurons (**A**–**C**, tdTomato*^Emx1^*) and SST neurons (**D**–**F**, tdTomato^SST^) were subjected to repeated isoflurane anesthesia at P15–P17 (2 hours a day for 3 consecutive days), and brains were fixed 7 days after the last anesthesia session (P24). Images of tdTomato under each condition are shown. (**C**) The number of tdTomato-positive cells per 4,000 μm^2^ in CA1 is presented for tdTomato*^Emx1^*. Control (*n* = 3) versus anesthesia (*n* = 4). *t* = 1.03; *P* = 0.35. (**E** and **F**) The number of tdTomato-positive cells is presented for tdTomato^SST^. (**E**) Hippocampus. Control (*n* = 4) versus anesthesia (*n* = 5). *t* = 1.38; *P* = 0.2. (**F**) Dentate gyrus. Control (*n* = 4) versus anesthesia (*n* = 5). *t* = 0.66; *P* = 0.52. Statistics are based on unpaired 2-tailed *t* test. Each data point represents an individual male animal. All data are presented as mean ± SEM. Scale bars: 100 μm (**A**, **D**); 50 μm (**B**).

To determine whether activation of the CC3-mediated apoptotic pathway in Pvalb neurons after repeated anesthesia in postnatal mice leads to a loss of Pvalb neurons, we quantified the number of fluorescent cells in tdTomato^Pvalb^ mice subjected to general anesthesia at P15–P17. The number of hippocampal Pvalb neurons was significantly reduced at day 7 after anesthesia (17.6% reduction at P24; Figure 1O) and remained low 43 days after anesthesia (21.8% reduction at P60; Figure 1P). No change in the total number of excitatory neurons (assessed in tdTomato^Emx1^ mice; Figure 2, A–C) or SST neurons (assessed in tdTomato^SST^ mice; Figure 2, D–F) was observed 7 days after anesthesia. Thus, repeated general anesthesia in juvenile mice leads to preferential apoptosis and the subsequent loss of Pvalb interneurons.

### Reduced synaptic inhibition of pyramidal neurons following repeated anesthesia.

**Figure 3:**
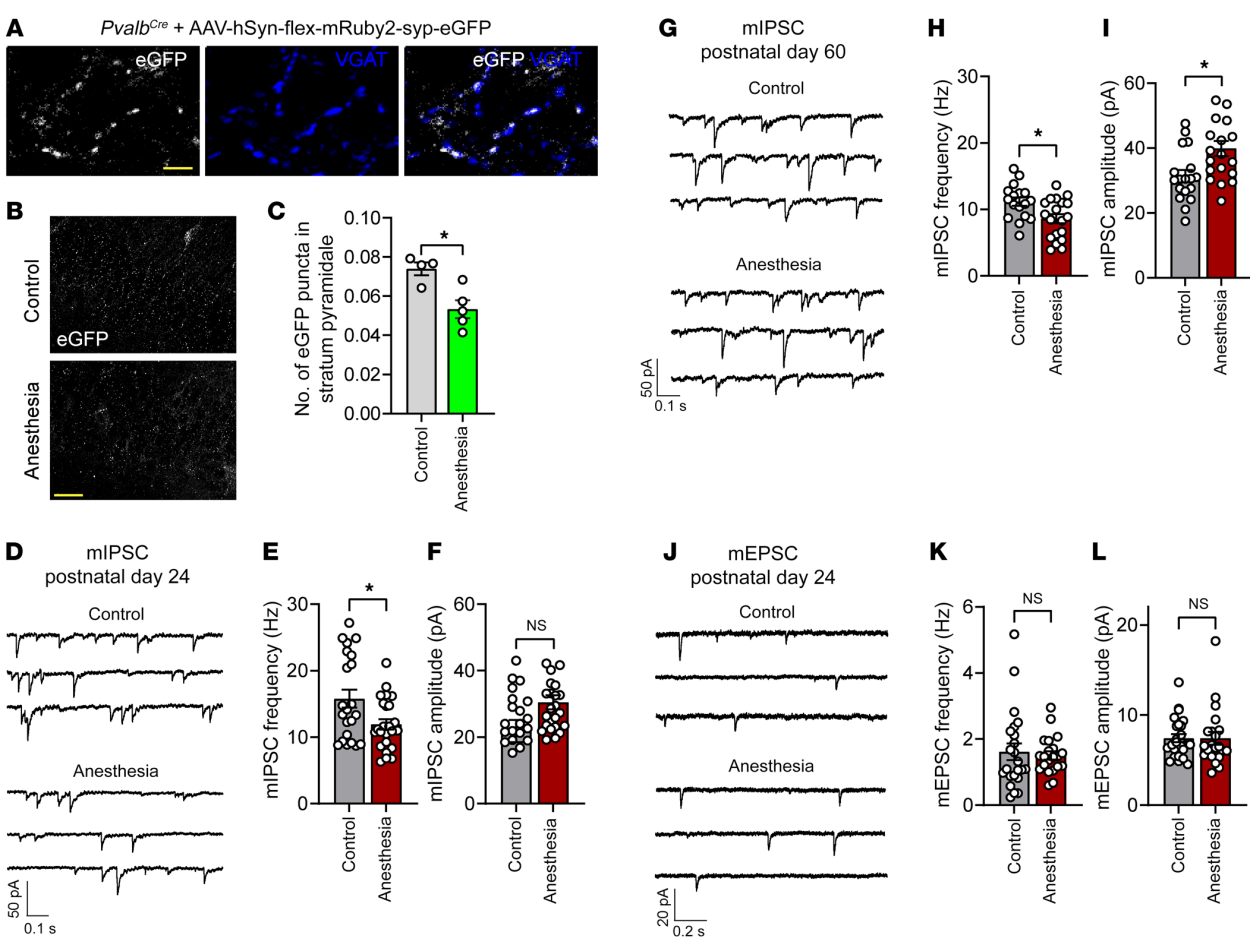
Repeated general anesthesia in juvenile mice reduces synaptic inhibition of hippocampal pyramidal neurons. (**A**) Synaptic contacts between Pvalb interneurons and pyramidal neurons in CA1 hippocampal area at P24 were labeled by injecting *Pvalb^Cre^* mice (i.c.v., P4) with AAV-hSyn-flex-mRuby2-syp-EGFP. EGFP puncta colocalized with staining for VGAT, conforming the labeling of inhibitory synaptic terminals. Scale bar: 5 μm. (**B**) The number of Pvalb inhibitory synaptic terminals (*Pvalb^Cre^* mice injected with AAV-hSyn-flex-mRuby2-syp-EGFP i.c.v. at P4) was significantly reduced at P24 in stratum pyramidale in male mice subjected to repeated anesthesia at P15–P17 as assessed by the number of EGFP puncta. Scale bar: 30 μm. (**C**) Control (*n* = 4) versus anesthesia (*n* = 5). *t*(7) = 3.44; *P* = 0.01, unpaired 2-tailed *t* test. (**D**) Representative traces of mIPSCs recorded from CA1 pyramidal neurons at P24 in male control mice and mice subjected to repeated general anesthesia at P15–P17. Frequency of mIPSC was decreased in mice subjected to repeated anesthesia. (**E**) Control (*n* = 21 cells from 9 mice) versus anesthesia (*n* = 23 cells from 8 mice). *t*(42) = 2.569; *P* = 0.0138, nested *t* test. mIPSC amplitude remained unchanged. (**F**) Control versus anesthesia. *t*(15) = 1.653; *P* = 0.1191, nested *t* test. (**G**) Recording of mIPSC in male mice 43 days after repeated anesthesia (P60) revealed decreased mIPSC frequency and increased mIPSC amplitude. (**H**) Control (*n* = 17 cells from 8 mice) versus anesthesia (*n* = 19 cells from 8 mice). *t*(34) = 2.713; *P* = 0.01, nested *t* test. (**I**) Control versus anesthesia. *t*(14) = 2.562, *P* = 0.023, nested *t* test. (**J**) Recording of mEPSCs at P24 showed repeated anesthesia at P15–P17 does not change mEPSC frequency or amplitude. (**K**) Control (24 cells from 8 mice) versus anesthesia (21 cells from 8 mice). *t*(43) = 0.941; *P* = 0.35, nested *t* test. (**L**) *t*(43) = 0.008, *P* = 0.99, nested *t* test. Each data point in **E**–**L** represents an individual cell. Data are represented as mean ± SEM. *P < 0.05.

Pvalb neurons make perisomatic inhibitory synapses on pyramidal neurons, powerfully suppressing their activity (22). To measure the number of synaptic contacts made by Pvalb neurons on pyramidal neurons, we injected *Pvalb^Cre^* mice with an adeno-associated virus (AAV) expressing synaptophysin EGFP in a Cre-dependent manner (AAV-hSyn-flex-mRuby2-syp-EGFP). We confirmed that the synaptophysin-EGFP puncta colocalize with VGAT (a marker of inhibitory presynaptic terminals), indicating specific labeling of presynaptic terminals (Figure 3A). Quantification of synaptophysin-EGFP puncta in CA1 stratum pyramidale revealed that the number of Pvalb presynaptic terminals was reduced at day 7 after anesthesia as compared with the control group (reduction of 27%; Figure 3, B and C). The number of excitatory presynaptic terminals, as assessed by the number of VGLUT1 puncta, remained unaltered (Supplemental Figure 3, A and B).

To determine functional changes in synaptic transmission, we performed patch-clamp recordings from hippocampal pyramidal neurons 7 days after anesthesia. Miniature inhibitory postsynaptic currents (mIPSC) showed a decrease in frequency (Figure 3, D and E), but not amplitude (Figure 3F), which is consistent with a loss of Pvalb neurons and a reduction in the number of Pvalb inhibitory terminals. The decrease in mIPSC frequency persisted at P60 (Figure 3, G and H), but at this age, an increase in mIPSC amplitude was detected (Figure 3I), likely representing a compensatory homeostatic response. No change in excitatory synaptic transmission was observed, as miniature excitatory postsynaptic current (mEPSC) frequency and amplitude remained unaltered (Figure 3, J–L). Together, these results show that the repeated general anesthesia–induced loss of Pvalb interneurons leads to a selective reduction of synaptic inhibition of pyramidal neurons.

### Inhibiting apoptosis in Pvalb neurons rescues memory impairment.

**Figure 4:**
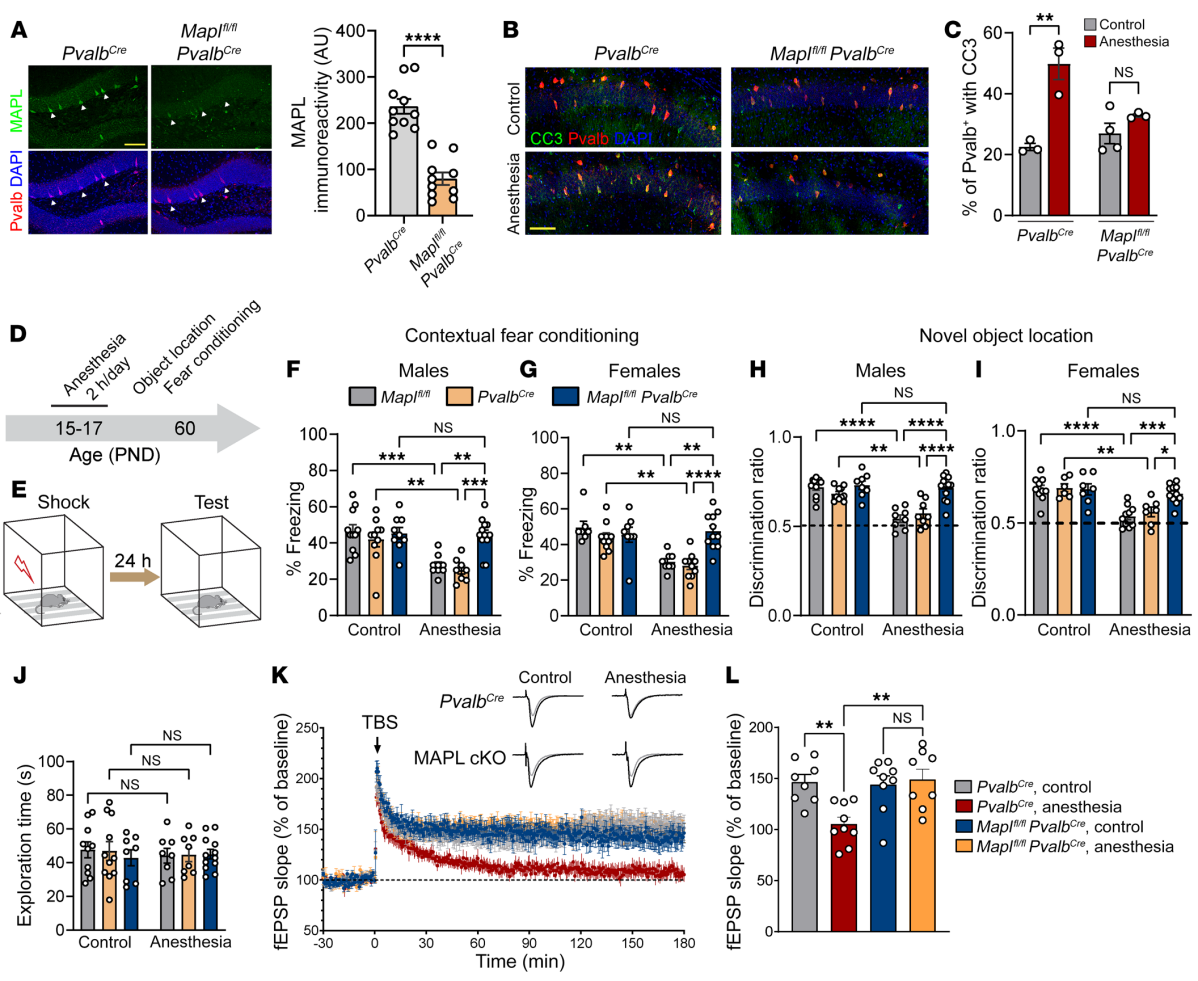
Blocking apoptosis in Pvalb neurons rescues repeated anesthesia-induced memory deficits in adulthood. (**A**) Immunostaining of hippocampal section. (Right) Quantification of MAPL levels in Pvalb neurons. (**B**) Repeated anesthesia in juvenile mice increases the number of Pvalb CC3^+^ cells 1 hour after the last anesthesia session in *Pvalb^Cre^* but not MAPL cKO^Pvalb^ mice. (**C**) Pvalb neurons were identified using IHC. Timeline of behavioral experiments (**D**) and schematic illustration of contextual fear conditioning (**E**). Long-term (24 hours after training) contextual fear memory was assessed in 2 control groups (*Mapl^fl/fl^* and *Pvalb^Cre^*) and *Mapl^fl/fl^* *Pvalb^Cre^* mice in males (**F**) and females (**G**). Whereas freezing behavior was reduced in control mice exposed to repeated anesthesia at P15–P17, there was no change in *Mapl^fl/fl^* *Pvalb^Cre^* mice. Novel object location test was performed in 2 control groups (*Mapl^fl/fl^* and *Pvalb^Cre^*) and *Mapl^fl/fl^* *Pvalb^Cre^* mice. Whereas object location memory was impaired in control mice exposed to repeated anesthesia at P15–P17, there were no deficits in *Mapl^fl/fl^* *Pvalb^Cre^* mice in males (**H**) and females (**I**). (**J**) Equal total exploration time. (**K**) Recording of fEPSP in CA1 area following TBS shows impaired L-LTP in *Pvalb^Cre^* mice subjected to repeated anesthesia at P15–P17, but not in MAPL cKO^Pvalb^ mice. Traces in gray represent baseline recording, whereas traces in black depict recording at 3 hours after TBS. (**L**) Quantification of mean potentiation 170 to 180 minutes after TBS. Statistics are based on 1-way (**C** and **L**) and 2-way (**F**–**I**) ANOVA followed by Tukey’s post hoc comparison. Scale bars: 100 μm. Each data point represents an individual animal. Data are represented as mean ± SEM. *P < 0.05; **P < 0.01; ***P < 0.001; ****P < 0.0001.

Repeated anesthesia in juvenile mice causes long-lasting deficits in long-term memory (1–3) and the late phase of long-term potentiation (L-LTP) (23), a putative cellular correlate of memory formation (24, 25). Since Pvalb neurons play important roles in memory consolidation (26, 27) and synaptic plasticity (28–30), we hypothesized that the loss of Pvalb neurons following repeated anesthesia in juvenile mice might underlie memory and L-LTP impairment. To test this hypothesis, we inhibited apoptosis selectively in Pvalb neurons before subjecting juvenile mice to anesthesia. We conditionally knocked out an apoptosis-mediating protein, MAPL (also called MUL1), in Pvalb neurons by crossing *Mapl^fl/fl^* mice (31) with *Pvalb^Cre^* mice (MAPL cKO^Pvalb^). MAPL SUMOylates the mitochondrial fission protein GTPase Drp1, which promotes apoptosis via the stabilization of ER-mitochondria contact sites, mitochondria fragmentation, and cytochrome *c* release (32). MAPL protein levels were reduced in Pvalb neurons in MAPL cKO^Pvalb^ mice (Figure 4A), and the ablation occurred postnatally as the expression of Cre recombinase under the control of the Pvalb promoter starts at the second postnatal week (Supplemental Figure 4, A and B), as previously reported (33–35). Consistent with the central role of MAPL in apoptosis (32), MAPL ablation in Pvalb neurons prevented repeated anesthesia-mediated induction of CC3 (P17; Figure 4, B and C) and the subsequent loss of Pvalb neurons (P24; Supplemental Figure 4, C and D). Consistent with this result, MAPL ablation also rescued the repeated anesthesia-induced reduction in the frequency of mIPSCs at P24 (Supplemental Figure 4, E–G).

To determine whether prevention of apoptosis in Pvalb neurons could rescue repeated anesthesia-induced memory impairments, we assessed memory formation in control and MAPL cKO^Pvalb^ mice. As expected with short-term anesthesia, a single 2-hour exposure of juvenile (P15) control mice to anesthesia did not impair long-term hippocampus-dependent memory in mature (P60) mice in contextual fear conditioning (Supplemental Figure 5A) and novel object location (Supplemental Figure 5, B and C) tasks. However, repeated exposures to general anesthesia led to deficits in long-term hippocampus-dependent memory, as previously reported (2, 19). Three consecutive 2-hour sessions of general anesthesia at P15–P17 led to deficits in both contextual fear conditioning and novel object location tests in P60 animals. Freezing behavior in contextual fear conditioning was decreased in repeated anesthesia-exposed control (*Mapl^fl/fl^* and *Pvalb^Cre^*) male and female mice at 24 hours after training as compared with nonanesthetized animals (experimental design, Figure 4, D and E; males, Figure 4F; females, Figure 4G). No change in freezing was observed immediately after training (Supplemental Figure 5, D and E), suggesting intact memory acquisition. Similarly, object location memory was impaired in male and female control mice subjected to repeated anesthesia at P15–P17 (males, Figure 4H; females, Figure 4I). No differences in total exploration time between the control and anesthesia-exposed mice were observed (Figure 4J). Remarkably, anesthesia-induced deficits in contextual fear conditioning were rescued in male and female mice lacking MAPL in Pvalb neurons (MAPL cKO^Pvalb^) (males, Figure 4F; females, Figure 4G). Likewise, impairments in novel object location memory in anesthesia-exposed animals were rescued in MAPL cKO^Pvalb^ male and female mice (Figure 4, H and I). A comparison between male and female mice showed no differences between the 2 sexes in the extent of repeated anesthesia-induced memory impairment and the rescue effect of MAPL ablation in Pvalb neurons (contextual fear conditioning, Supplemental Figure 6A; novel object location, Supplemental Figure 6B). To study whether the repeated anesthesia-induced memory impairment persists after the age of 2 months, we subjected new cohorts of male and female mice to repeated anesthesia at P15–P17 and tested their long-term memory four and a half months later (at the age of 5 months). We found that memory was impaired in repeated anesthesia-exposed 5-month-old control (*Mapl^fl/fl^* and *Pvalb^Cre^*) male and female animals in both contextual fear conditioning (Supplemental Figure 6C) and novel object location (Supplemental Figure 6D) tasks. MAPL cKO^Pvalb^ were protected from anesthesia-induced deficits in both sexes (Supplemental Figure 6, C and D; comparison between males and females in Supplemental Figure 6, E and F).

We also assessed repeated anesthesia-induced memory deficits in mice with deletion of MAPL in excitatory neurons (*Mapl^fl/fl^* *Emx1^Cre^*). Anesthesia-exposed *Mapl^fl/fl^* *Emx1^Cre^* mice (P15–P17) were not protected from anesthesia-induced memory defects, as they exhibited impairments in both contextual fear conditioning (Supplemental Figure 7A) and object location (Supplemental Figure 7, B and C) tasks at P60. Thus, apoptosis and loss of Pvalb neurons following repeated anesthesia in juvenile mice substantially contribute to long-lasting memory impairment as selective prevention of Pvalb neurons apoptosis corrects memory deficits in adulthood.

We next investigated whether inhibition of apoptosis in Pvalb neurons also protects against repeated anesthesia-induced defects in L-LTP. Field potential recordings in the hippocampal CA1 area showed that the theta-burst stimulation–induced (TBS-induced) L-LTP was reduced in control 8-week-old mice subjected to repeated general anesthesia at P15–P17 (Figure 4, K and L). Consistent with behavioral experiments, L-LTP remained intact in anesthesia-exposed MAPL cKO^Pvalb^ animals (Figure 4, K and L), indicating that these mice are resistant to anesthesia-induced defects in synaptic plasticity. Collectively, our results demonstrate that blocking Pvalb neuron loss following repeated anesthesia in postnatal mice rescues anesthesia-induced persistent deficits of pyramidal cell inhibition and synaptic plasticity as well as hippocampus-dependent memory.

### Ablation of Pvalb neurons causes long-lasting memory deficits.

**Figure 5:**
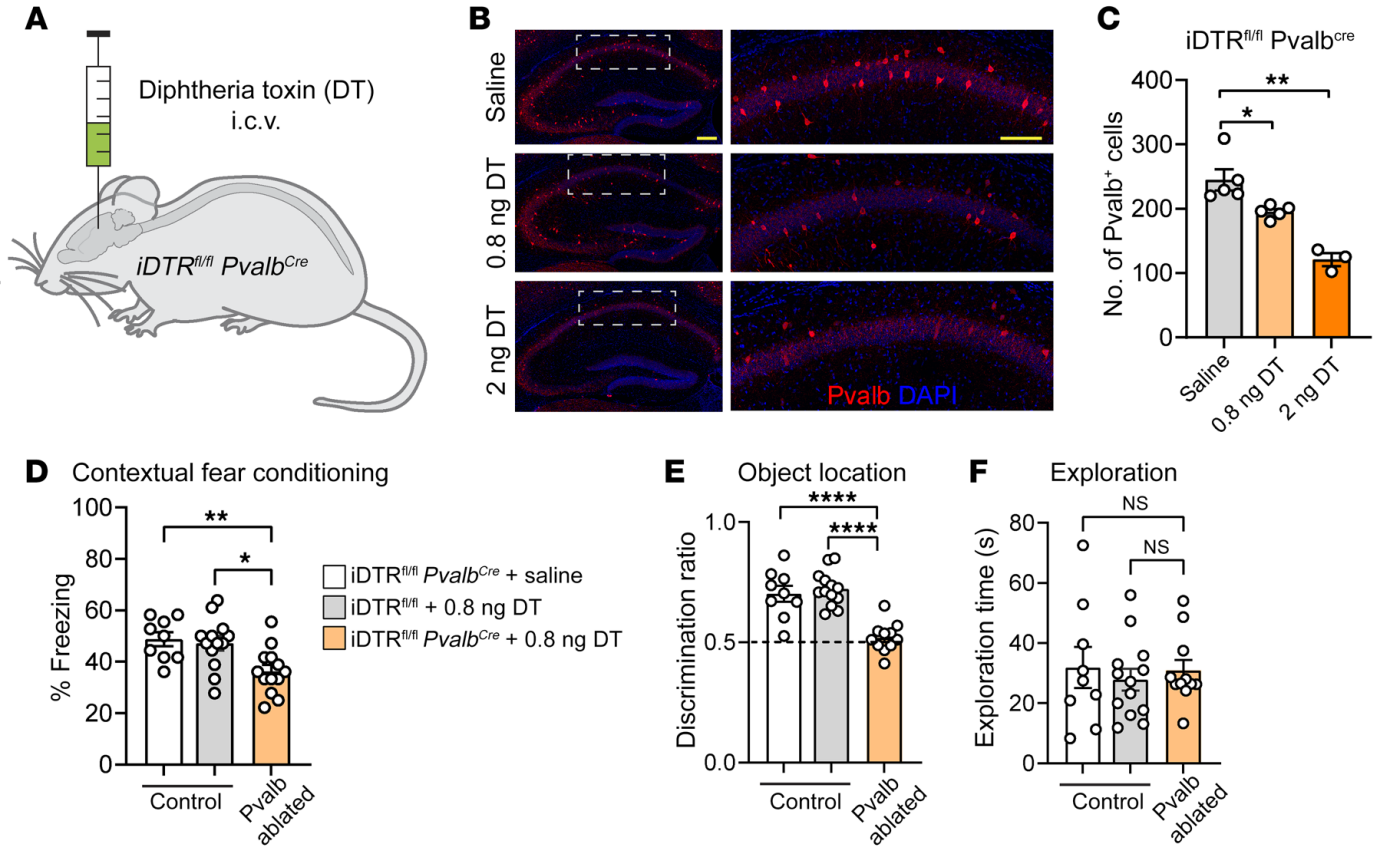
Ablation of Pvalb neurons in juvenile mice causes persistent memory deficits. (**A**) Male mice expressing DT receptor in Pvalb neurons (iDTR*^fl/fl^* *Pvalb^Cre^*) were injected i.c.v. with increasing doses of the DT at P16. (**B**) Immunohistochemistry against Pvalb reveals a dose-dependent decrease in the number of hippocampal Pvalb neurons in DT-injected mice. (**C**) Saline (*n* = 5 mice) versus 0.8 ng DT (*n* = 5 mice), *q*(10) = 4.397; *P* = 0.0273. Saline versus 2 ng DT (*n* = 3 mice), *q*(10) = 9.751; *P* = 0.0066. Statistics are based on 1-way ANOVA followed by Tukey’s post hoc comparison. Scale bars: 200 μm (left); 100 μm (right). (**D**) Contextual fear conditioning (24 hours after training) shows reduced freezing behavior in mice with ablation of Pvalb neurons as compared with mice in 2 control groups. iDTR*^fl/fl^* *Pvalb^Cre^* + saline (*n* = 9) versus iDTR*^fl/fl^* *Pvalb^Cre^* + 0.8 ng DT (*n* = 13), *q*(32) = 4.435; *P* = 0.01. iDTR*^fl/fl^* + 0.8 ng DT (*n* = 13) versus iDTR*^fl/fl^* *Pvalb^Cre^* + 0.8 ng DT (*n* = 13), *q*(32) = 4.295; *P* = 0.0128. Statistics are based on 1-way ANOVA followed by Tukey’s post hoc comparison. (**E**) Object location memory (24 hours after training) was impaired in mice with partial ablation of Pvalb neurons, but not in mice in control groups. iDTR*^fl/fl^* *Pvalb^Cre^* + saline (*n* = 9) versus iDTR*^fl/fl^* *Pvalb^Cre^* + 0.8 ng DT (*n* = 13), *q*(30) = 7.39; *P* < 0.001. iDTR*^fl/fl^* + 0.8 ng DT (*n* = 11) versus iDTR*^fl/fl^* *Pvalb^Cre^* + 0.8 ng DT (*n* = 13), *q*(30) = 9.001; *P* < 0.001. Statistics are based on 1-way ANOVA followed by Tukey’s post hoc comparison. (**F**) All 3 groups show equal total exploration (*P* > 0.05). Each data point represents an individual animal. All data are represented as mean ± SEM. *P < 0.05, **P < 0.01, ****P < 0.0001.

To further study the role of Pvalb neurons in long-lasting cognitive impairment, we investigated whether loss of Pvalb neurons in postnatal mice is sufficient to recapitulate persistent memory deficits observed after repeated anesthesia. To this end, we ablated Pvalb neurons in juvenile mice by injecting diphtheria toxin (DT) into mice expressing the DT receptor in Pvalb neurons (iDTR;*Pvalb^Cre^*, Figure 5A). An i.c.v. injection of increasing doses of DT at P16 caused a dose-dependent ablation of Pvalb neurons in the hippocampus at P24 (Figure 5, B and C). To mimic the anesthesia-induced loss of Pvalb neurons, we injected iDTR;*Pvalb^Cre^* mice (i.c.v.) with 0.8 ng of DT at P16, which caused a reduction in the number of Pvalb neurons in the hippocampus comparable to that seen with repeated anesthesia (20.4% reduction; Figure 5C). As controls, we used iDTR;*Pvalb^Cre^* mice injected with saline or iDTR mice injected with the same dose of DT (0.8 ng). Behavioral experiments at P60 revealed that mice with partial ablation of Pvalb neurons exhibited deficits in contextual fear conditioning (24 hours after training; Figure 5D) and novel object location (Figure 5E) tests as compared with control animals. No differences in total exploration in the object-location test were observed between the groups (Figure 5F). These results demonstrate that the loss of Pvalb neurons in juvenile mice is sufficient to cause long-lasting memory deficits.

## Discussion

We identified Pvalb inhibitory interneurons as a vulnerable cell population that undergoes preferential apoptosis following repeated anesthesia in juvenile mice and showed that loss of Pvalb neurons engenders long-lasting impairment in synaptic inhibition of pyramidal cells and hippocampus-dependent memory.

Previous studies have suggested that apoptosis following postnatal repeated anesthesia contributes to memory deficits in adult animals (1, 3). However, the causal relationships between neural apoptosis and memory impairment have not been established. Additionally, an understanding of whether specific neuronal subpopulations are preferentially affected and how the loss of only a small fraction of neurons induces long-lasting cognitive defects has remained elusive. Our results identify Pvalb neurons as a neuronal population that is preferentially affected by repeated anesthesia. We show that the loss of Pvalb neurons is necessary and sufficient to induce persistent hippocampus-dependent memory deficits. Since each Pvalb neuron regulates the activity of more than a hundred pyramidal neurons (22) and Pvalb neurons are involved in memory consolidation via coordinating and stabilizing CA1 network dynamics (26) and mediating hippocampal-neocortical interactions following training (27, 36), our findings provide a plausible explanation as to why the loss of a small number of neurons after repeated anesthesia results in a robust and persistent memory impairment.

Previous studies have suggested increased sensitivity of Pvalb neurons to cellular stress, particularly during the early postnatal period when they undergo maturation and acquire molecular and phenotypic identity (37). The increased vulnerability of Pvalb as compared with other inhibitory and excitatory neurons is likely related to their unique fast-spiking activity pattern and innervation of numerous excitatory neurons by an extensive axonal arbor. These properties impose a high metabolic demand requiring increased energy production in Pvalb neurons, which have significantly greater mitochondrial density as compared with other neuronal cell types (38). This metabolic demand might exhaust the adaptive homeostatic mechanisms under repeated anesthesia conditions that cause oxidative stress, triggering proapoptotic signaling and cell death. An additional reason for the sensitivity of Pvalb neurons to repeated anesthesia in neonates might be related to the lack of perineuronal nets (PNNs) around Pvalb neurons at this age. PNNs are extracellular structures formed around Pvalb neurons during the fourth postnatal week in mice (39). PNNs, which protect Pvalb neurons from oxidative stress (40, 41), are not yet fully formed around Pvalb neurons during repeated general anesthesia at P15–P17, potentially making Pvalb neurons more vulnerable. Future studies should decipher the molecular mechanisms underlying the increased sensitivity of Pvalb neurons to repeated anesthesia in neonates and develop strategies rendering Pvalb neurons more resilient to cellular stress during repeated anesthesia.

The selection of our repeated anesthesia protocol (3 consecutive 2-hour sessions at P15–P17) was based on previous studies in animal models mimicking repeated exposure of children to general anesthesia (18, 19). Mice at P15 have been equated to 1- to 3-year-old children based on weaning age and life span (42). This is the age when many repeated procedures under anesthesia are performed in the pediatric population (43). Since Pvalb neurons mature and acquire their identity during the second postnatal week in rodents, their investigation during the first postnatal week, which is a commonly used period in studies of repeated anesthesia in preclinical models, is challenging. Exposure of mice to anesthesia at P15–P17, when a substantial portion of Pvalb neurons have already acquired their identity and express Pvalb, allowed us to identify Pvalb neurons as a vulnerable subpopulation to apoptosis.

In this work, we focused on the hippocampus since this brain structure, which is critical for several types of memory (42, 44), has been shown to exhibit anesthesia-induced apoptosis in different species (1). Additionally, hippocampus-dependent memory tests allowed us to establish the causal link between hippocampal apoptosis and area-specific memory formation. Notably, increased apoptosis after repeated anesthesia was found in all hippocampal areas, except the subiculum. It remains to be determined why the subiculum, which is the primary hippocampal output structure harboring distinct cellular populations and neuronal circuits (45–47), was not affected. Consistent with previous studies (48, 49), we found anesthesia-induced apoptosis in the cortex and revealed substantial apoptosis of cortical Pvalb neurons. Future studies should assess the role of Pvalb neuron apoptosis in modulating cortical plasticity and examine whether similar mechanisms occur in other brain regions (1).

Repeated anesthesia in juvenile mice induced long-lasting memory deficits in both sexes. Moreover, MAPL ablation in Pvalb neurons rescued memory deficits in male and female mice, suggesting similar underlying mechanisms. Nevertheless, performing molecular analyses in only males is a limitation of the study, requiring confirmation of our conclusions in female animals.

In this work, we demonstrate a critical role for Pvalb neuron apoptosis in long-lasting hippocampus-dependent memory deficits following anesthesia in young mice. Additional mechanisms, however, may also contribute to the long-lasting cognitive impairment, including decreased neurogenesis (2) and dysfunction of nonneuronal cells (50).

In summary, our work identifies Pvalb neurons as a subpopulation susceptible to apoptosis during repeated general anesthesia in juvenile mice and shows that loss of Pvalb neurons mediates memory deficits in adulthood.

## Methods

### Animals and environment.

C57BL/6 mice were bred in-house at the McGill University animal facility. To generate mice with cell-type–specific expression of tdTomato, *tdTomato* reporter mice (Ai14, The Jackson Laboratory [JAX], stock 007914) were crossed with mice expressing Cre under the *GAD2* promoter (*GAD2^Cre^*, JAX, stock 010802), SST promoter (*SST^Cre^*, JAX, stock 013044), Pvalb promoter (*Pvalb^Cre^*, JAX, stock 008069), *VIP* promoter (*VIP^Cre^*, JAX, stock 010908), and *Emx1* promoter (*Emx1^Cre^*, JAX, stock 005628). To generate MAPL conditional knockout (cKO) animals, *Mapl^loxP/loxP^* mice (*Mapl^fl/fl^*, described previously, ref. 31) were crossed with *Pvalb^Cre^* mice to generate *Mapl^fl/WT^ Pvalb^Cre^* mice. These mice were bred with each other to generate experimental *Mapl^fl/fl^ Pvalb^Cre^* cKO mice, control *Mapl^fl/fl^* mice, and control *Pvalb^Cre^* mice. Using the same breeding strategy, we bred *Mapl^fl/fl^* mice with *Emx1^Cre^* mice to generate *Mapl^fl/fl^ Emx1^Cre^* mice and 2 control groups (*Mapl^fl/fl^* and *Emx1^Cre^*). Cre-inducible diphtheria receptor mice (ROSA26iDTR, JAX, stock 007900) were crossed with *Pvalb^Cre^* mice to generate iDTR;*Pvalb^Cre^* mice. All mice were on a C57BL/6J genetic background. Mice were housed in standard shoebox cages (4–5 mice in each cage) and kept on a 12-hour light/12-hour dark cycle (lights on at 0700 hours). Food and tap water were provided ad libitum. Mice were maintained at the McIntyre Medical Building and Goodman Cancer Research Center in ventilated, temperature- and humidity-controlled rooms. Behavioral experiments shown in Figure 4 and Supplemental Figure 6 were conducted on male and female mice, whereas molecular and electrophysiology experiments and the behavioral experiments shown in Figure 5 were performed using male animals. Sample sizes were determined based on previous behavioral, molecular, and electrophysiological data published by our laboratories (18). In all experiments, animals were randomly assigned to treatment groups. The experimenter was blinded to the genotype and condition during data acquisition and analysis in all studies.

### Anesthesia administration.

Repeated general anesthesia was performed during P15–P17 (3 days in total) for 2 hours a day using 1.5% isoflurane (AErrane, Baxter, in 100% oxygen), which is comparable to pediatric minimum alveolar concentration (MAC) of 1.8%. The airflow rate was 2 L/min. The animals were anesthetized in a bottom-heated chamber (8 × 4 × 5 inches), and protective eye gel (Systane Ointment) was applied at the beginning of each anesthesia session. Animals were turned over every 15 minutes. The breathing pattern was monitored every 5 minutes for the first 15 minutes and every 10 minutes thereafter. The depth of anesthesia was adjusted accordingly. At the end of the anesthesia session, mice were allowed to recover in a bottom-heated cage. RR, sPO_2_%, and HR were monitored continuously (MouseOx Plus, Starr Life Sciences Corp.), and data were recorded every 5 minutes.

### Novel object location.

Adult (8 week old and 5 month old) male and female mice were used. All experiments were performed by a researcher blinded to experimental conditions and genotype. Object location memory testing was run over 6 days. Days 1 and 2 included a morning and afternoon handling session (1 minute per mouse in the behavior room). Day 3 consisted of a 10-minute morning habituation session in an empty arena (arena dimension, 60 × 60 × 30 cm). Day 4 (P60) and day 5 involved two 10-minute training sessions (morning and afternoon) separated by 4 hours, with similar objects placed in the middle between the corners close to the wall (opposite walls for 2 objects), for a total of 4 training sessions. Testing (10 minutes) was performed in the afternoon of day 6 (24 hours after the last training session), where 1 object was randomly moved to a corner of the arena. The mice were recorded with a video camera. The discrimination ratio was calculated as time spent exploring the moved object over the total time exploring both objects. Mice exploring for less than 10 seconds were excluded.

### Contextual fear conditioning.

Adult (8 week old and 5 month old) male and female mice were used. Contextual fear conditioning was performed on the mice (P63 and postnatal 5 months) that underwent the novel object location test previously. The training protocol consisted of a 2-minute period of context exploration, followed by two 1-second 0.6 mA foot shocks separated by 60 seconds. The mice were returned to their home cage 1 minute after the second foot shock. The mice were tested for contextual fear memory by placing them 24 hours later in the training chamber for 3 minutes. The incidence of freezing was scored in 5-second intervals as either “freezing” or “not freezing.” Percentage of freezing indicates the number of intervals in which freezing was observed divided by the total number of 5-second intervals.

### Field potential recordings.

Male mice (8 weeks old) were anesthetized with isoflurane, and the brain was rapidly removed and placed in ice-cold oxygenated artificial cerebrospinal fluid (aCSF) containing 124 mM NaCl, 2.5 mM KCl, 1.25 mM NaH_2_PO_4_, 1.3 mM MgSO_4_, 2.5 mM CaCl_2_, 26 mM NaHCO_3_, and 10 mM glucose. Transverse hippocampal slices (400 μm), prepared using a Leica VT1200S Vibratome, were allowed to recover submerged for at least 2 hours at 32°C in oxygenated aCSF and for an additional 30 minutes in a recording chamber at 27–28°C while perfused with aCSF.

Field extracellular postsynaptic potentials (fEPSPs) were recorded in the CA1 stratum radiatum with glass electrodes (2–3 MΩ) filled with aCSF. Schaffer collateral fEPSPs were evoked by stimulation with a concentric bipolar tungsten electrode placed in the midstratum radiatum proximal to the CA3 region. Baseline stimulation was applied at 0.033 Hz by delivering 0.1 ms pulses, with intensity adjusted to evoke 35% of maximal fEPSPs. TBS consisted of 15 bursts of 4 pulses at 100 Hz separated by 200 ms intervals. The experiments were performed by a researcher blinded to the experimental condition and genotype.

### Whole-cell patch-clamp recording.

Male mice were anesthetized by quick exposure to isoflurane, and coronal hippocampal slices (300 μm) were cut using a Vibratome (Leica 2100S) in an ice-cold solution containing 75 mM sucrose, 87 mM NaCl, 2.5 mM NaH_2_PO_4_, 1.25 mM MgSO_4_, 0.5 mM CaCl_2_, 25 mM glucose, and 25 mM NaHCO_3_. Slices were transferred to aCSF containing 124 mM NaCl, 2.5 mM KCl, 1.25 mM NaH_2_PO_4_, 24 mM NaHCO_3_, 2 mM MgCl_2_, 2 mM CaCl_2_, and 12.5 mM glucose. Whole-cell recordings were obtained from CA1 pyramidal neurons using patch pipettes (borosilicate glass capillaries; 3–5 MΩ). The intracellular solution to record mEPSCs contained 120 mM CsMeSO_4_, 5 mM CsCl, 2 mM MgCl_2_, 10 mM HEPES, 0.5 mM ethylene glycol-bis(β-aminoethyl ether)-N,N,N′,N′-tetraacetic acid (EGTA), 2 mM QX-314, 10 mM Na_2_-phosphocreatine, 2 mM ATP-tris, and 0.4 mM GTP-tris. Tetrodotoxin (TTX) (1 μM, Abcam) and Gabazine (5 μM, Tocris) were added to the extracellular fluid. To record mIPSCs, the intracellular solution contained 135 mM CsCl, 10 mM HEPES, 2 mM QX-314, 2 mM MgCl_2_, and 4 mM MgATP. TTX (1 μM), D-APV (50 μM, Abcam), and DNQX (5 μM, Abcam) were added to the extracellular fluid. Slices were placed in a recording chamber at 28 to 30°C, and mEPSCs and mIPSCs were obtained in voltage-clamp mode using a Multiclamp 700A amplifier (Molecular Devices) at –70 mV and detected by pClamp 10 software (Molecular Devices).

Series resistance was routinely monitored. Recorded signals were low-pass filtered at 2 kHz and digitized at 20 kHz. Data were included only if the holding current were stable or if series resistance varied by less than 25% of the initial value.

### DT administration.

DT (Sigma, D0564) was administered via freehand i.c.v. injection (0.4 μl per ventricle at 1 ng/μL) as previously described (51, 52). P16 pups were anesthetized with 2% isoflurane for 3 minutes and placed in a sternal recumbent position. Using standard aseptic technique, a Hamilton 1701RN 10 μL syringe was fitted with a 2-inch 26-gauge needle. Stiff tubing was placed to expose 2.5 mm of the needle from the bevel tip in order to standardize injection depth. The location of the ventricles was identified by drawing a point midline between the anterior base of the ears and feeling for the bregma with the needle tip. The needle was inserted at a 45-degree angle 2 mm below and 2 mm lateral to bregma, and 0.4 μl of the DT solution was injected slowly into each ventricle.

### AAV injection.

AAV-hSyn-flex-mRuby2-syp-EGFP, produced by the Canadian Neurophotonics Platform Viral Vector Core Facility (RRID:SCR_016477) was injected i.c.v. into P4 *Pvalb^Cre^* mice via freehand technique as described previously (53). P4 pups underwent hypothermia-induced anesthesia, as follows: a flat aluminum plate on top of the ice was used as the injection surface, and a dry task wipe was placed to protect neonatal skin; pups were then placed on the surface for 3 minutes until fully anesthetized. Lambda and bregma sutures are visible and serve as landmarks for freehand i.c.v. injection. The injection site (approximately 2/5 distance between lambda suture and the eye) was marked with a laboratory pen. A Hamilton 1701RN 10 μL syringe fitted with a 2-inch 26-gauge needle filled with AAV was inserted at a depth of 2.5 mm, and 1 μL of the virus was slowly injected into each ventricle. The pups were allowed to recover on a warming pad.

### Immunohistochemistry and image analysis.

Mice were sacrificed by intracardiac perfusion with 4% paraformaldehyde at predetermined time points (P14, P17, P24, or P60). Brains from mice at P0 and P7 were fixed without cardiac perfusion (drop-fix into 4% paraformaldehyde). The brains were post-fixed at 4°C, and 50 μm thick sequential coronal sections were taken from the beginning to the end of the dorsal hippocampus using a Leica VT1200S Vibratome. Three sequential hippocampal sections, 200 μm apart, from both dorsal hippocampi per mouse were used for immunohistochemistry. Sections were washed 3 times for 5 minutes with 0.2% Triton X-100 in PBS in a shaker. Sections were permeabilized and blocked with 10% goat serum in PBS Triton-X 0.2% (PBST) for 1 hour, then incubated with the primary CC3 antibody (1:100 in PBST, STJ 97448), primary Pvalb antibody (1:500 in 5% goat serum in PBST, Synaptic Systems 195 004), primary VGAT antibody (1:1,000 in 5% goat serum in PBST, Synaptic Systems 131 002), primary VGLUT1 antibody (1:500 in 5% goat serum in PBST, Millipore AB5905), and primary MAPL/MUL1 antibody (1:50 in 5% goat serum in PBST, MilliporeSigma HPA017681) at 4°C overnight, washed 3 times in PBS, and incubated with the secondary antibody (Alexa Fluor 488, Alexa Fluor 568, or Alexa Fluor 647, 1:500 in PBS, Thermo Fisher Scientific) for 2 hours. Sections were washed once with PBST, then PBS alone, and finally with PBS and DAPI at a 1:5,000 dilution in PBS. The sections were mounted on microscope coverslips using Invitrogen ProLong Gold Antifade Reagent (Thermo Fischer Scientific), and images were acquired with a Zeiss epifluorescence microscope equipped with Apotome using a ×20 objective. Synapse images were acquired with Airyscan microscopy using a Zeiss ×63/1.40 Oil DIC f/ELYRA objective and the Airyscan super-resolution (SR) module with a 32-channel hexagonal array GaAsP detector on LSM880 (Zeiss). The number of CC3-positive cells or neuronal marker–positive (GAD2, Pvalb, SST, VIP, EMX1) cells per section were counted and averaged between both dorsal hippocampi (left and right, all hippocampal areas). Three sections per mouse were analyzed for CC3-positive cells or neuronal marker–positive cells by a researcher blinded to the experimental condition or genotype, and the sum of positive cells in 3 sections was calculated and presented. To count synaptic puncta using ImageJ (NIH), all images were converted to 8 bit and threshold levels were standardized for all sections. Quantification was performed in the same region of interest (ROI) (2,800 μm^2^) for all sections. For each hippocampus, 3 measurements of synaptic puncta were taken for every area of interest (stratum oriens, stratum pyramidale, and stratum radiatum) and the average reported.

### Statistics.

All results are expressed as mean ± SEM. All statistical tests (GraphPad Prism 7.03) were performed using Student’s 2-tailed *t* test or 1-way or 2-way ANOVA, as appropriate, followed by between-group comparisons using Tukey’s post hoc test, with α = 0.05 as the significance criteria.

### Study approval.

All procedures complied with the Canadian Council on Animal Care guidelines and were approved by McGill University’s downtown Animal Care Committee.

## Author contributions

PSR, T Schricker, LW, HS, T Sato, CGG, JSM, JCL, MPK, HMM, and AK conceived the project, designed experiments, and supervised the research. PSR performed all behavioral and IHC experiments with the help of NB, ND, LNL, MH, CW, SH, KCL, and CS. CTP and MJE performed electrophysiological recordings under the supervision of JCL. VMG and HMM generated *Mapl^fl/fl^* mice. SH, MDI, KN, and VS assisted with the design of behavioral studies. PSR and AK wrote the manuscript. All authors reviewed the manuscript and discussed the work.

## Supplementary Material

Supplemental data

## Version 1 11/17/2022

In-Press Preview

## Version 2 01/17/2023

Electronic publication
