# Supplementary material for: Parvalbumin interneuron loss mediates repeated anesthesia-induced memory deficits in mice
Source: J Clin Invest. 2023 Jan 17;133(2):e159344. doi: 10.1172/JCI159344 (PMC9843048; doi:10.1172/JCI159344)
Supplement: Supplemental data [file jci-133-159344-s059.pdf]

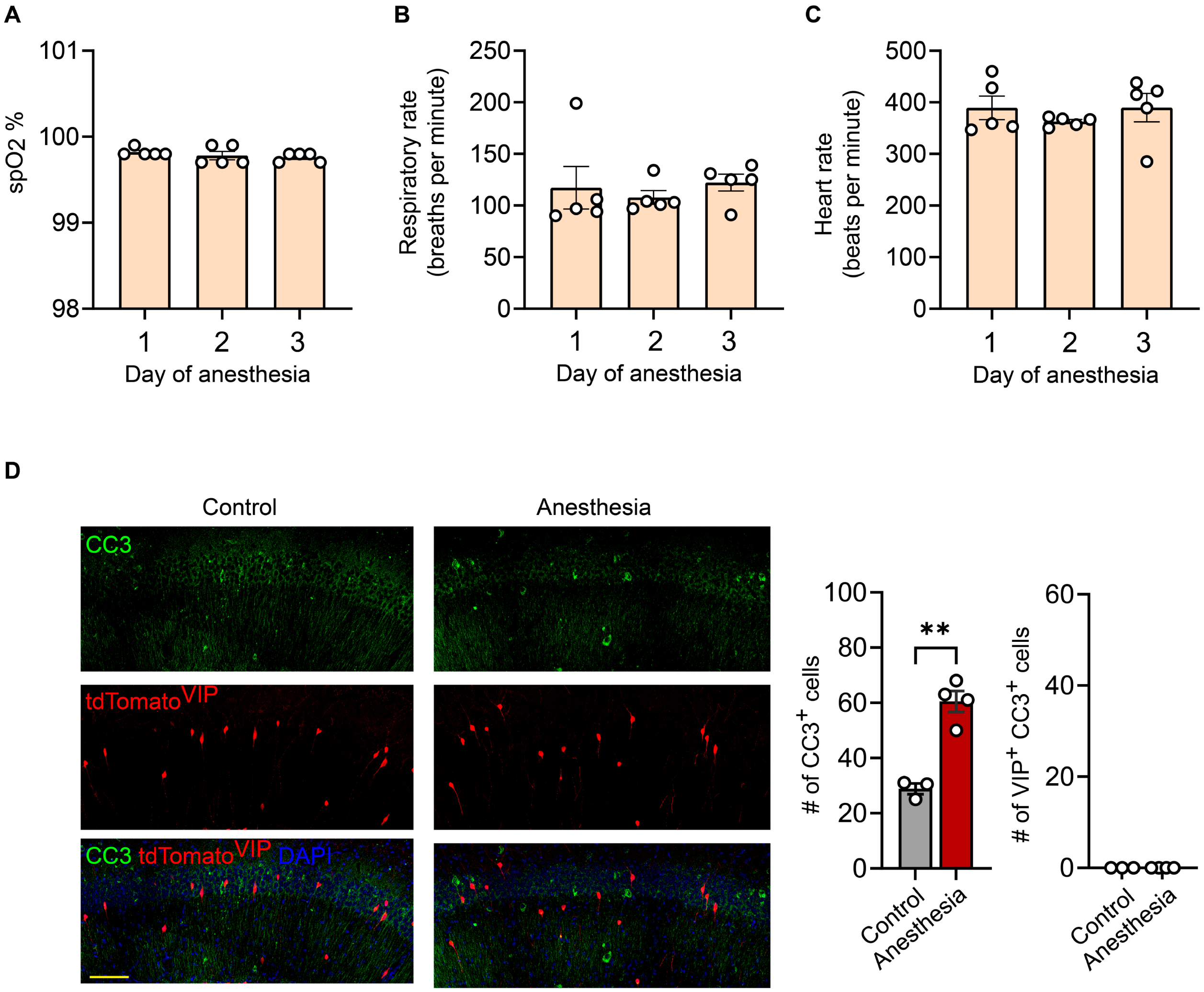

Supplemental Figure 1. Stable hemodynamic state of juvenile mice subjected to repeated general anesthesia.
Oxygen saturation levels (SpO_2, **A**), respiratory rate (**B**), and heart rate (**C**) remained stable during the three days of anesthesia (n = 5/group). No oxygen desaturation event (defined as an SpO_2 < 92%) occurred at any time while the mice were anesthetized. No differences were found between groups in repeated measure ANOVA. (**D**) Apoptotic marker CC3 does not co-localizes with VIP neurons. Immunostaining of hippocampal sections for CC3 in $TdTomato^{VIP}$ mice revealed no co-localization of CC3-positive neurons with VIP neurons (right graph), while the total number of CC3-positive cells was significantly increased (n = 3/group, $t = 5.525$, $p = 0.0052$, Student's *t*-test, two-tailed) post-anesthesia (left graph). Scale bar, 100 µm. Each data point represents individual animal (male + female). Data are presented as mean ± s.e.m.

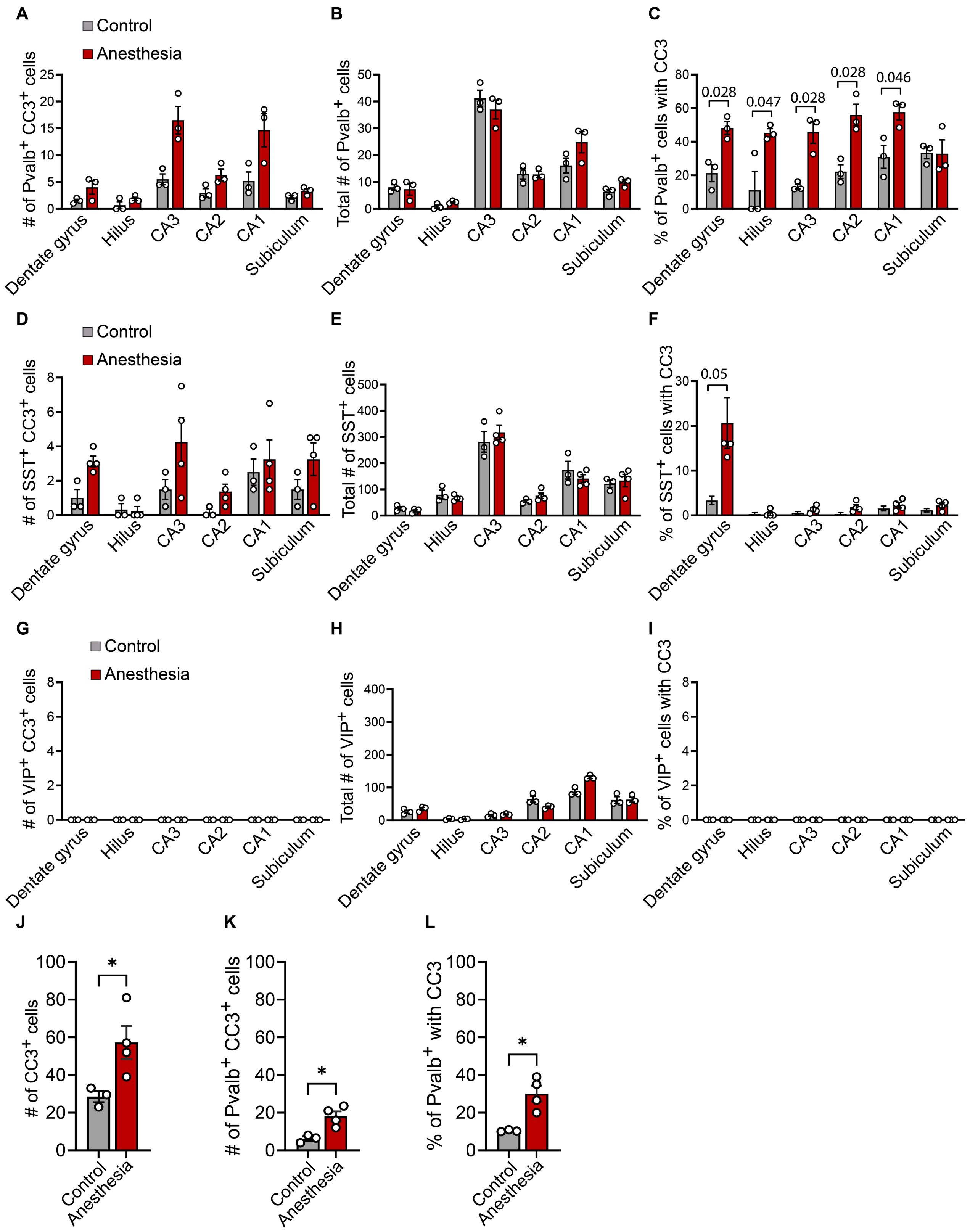

Supplemental Figure 2. Distribution of repeated anesthesia induced-CC3 in Pvalb, SST and VIP neurons in different hippocampal regions.

Mice expressing tdTomato in Pvalb (**A**-**C**), SST (**D**-**F**) and VIP (**G**-**I**) neurons were subjected to repeated anesthesia at PD 15-17 and brains were extracted one hour after the last anesthesia session and processed for CC3 immunostaining. Quantification shows the number of Pvalb $CC3^+$ neurons (**A**), total number of Pvalb neurons (**B**), and proportion of all Pvalb neurons showing CC3 immunoreactivity (**C**, Pvalb $CC3^+$/ total number of Pvalb neurons) in different hippocampal areas. The number of SST $CC3^+$ neurons (**D**), total number of SST neurons (**E**), and proportion of SST neurons with CC3 (**F**) in different hippocampal areas are shown. The number of VIP $CC3^+$ neurons (**G**), total number of VIP neurons (**H**), and proportion of VIP neurons with CC3 (**I**) in different hippocampal areas are presented. (**J**-**L**) Mice expressing tdTomato in Pvalb neurons were subjected to repeated anesthesia at PD 15-17 and brains were extracted one hour after the last anesthesia session and processed for CC3 immunostaining in the cortex (primary somatosensory area). The number of $CC3^+$ neurons (**J**), number of $Pvalb^+$ $CC3^+$ neurons (**K**), and proportion of Pvalb neurons with CC3 (**L**) in the cortex (per 2.83 mm^2) are shown. Statistics are based on unpaired 2-tailed *t*-test in all panels. The *p* value is shown for conditions with significant differences. Each data point represents individual male animal. All data are presented as mean ± s.e.m.

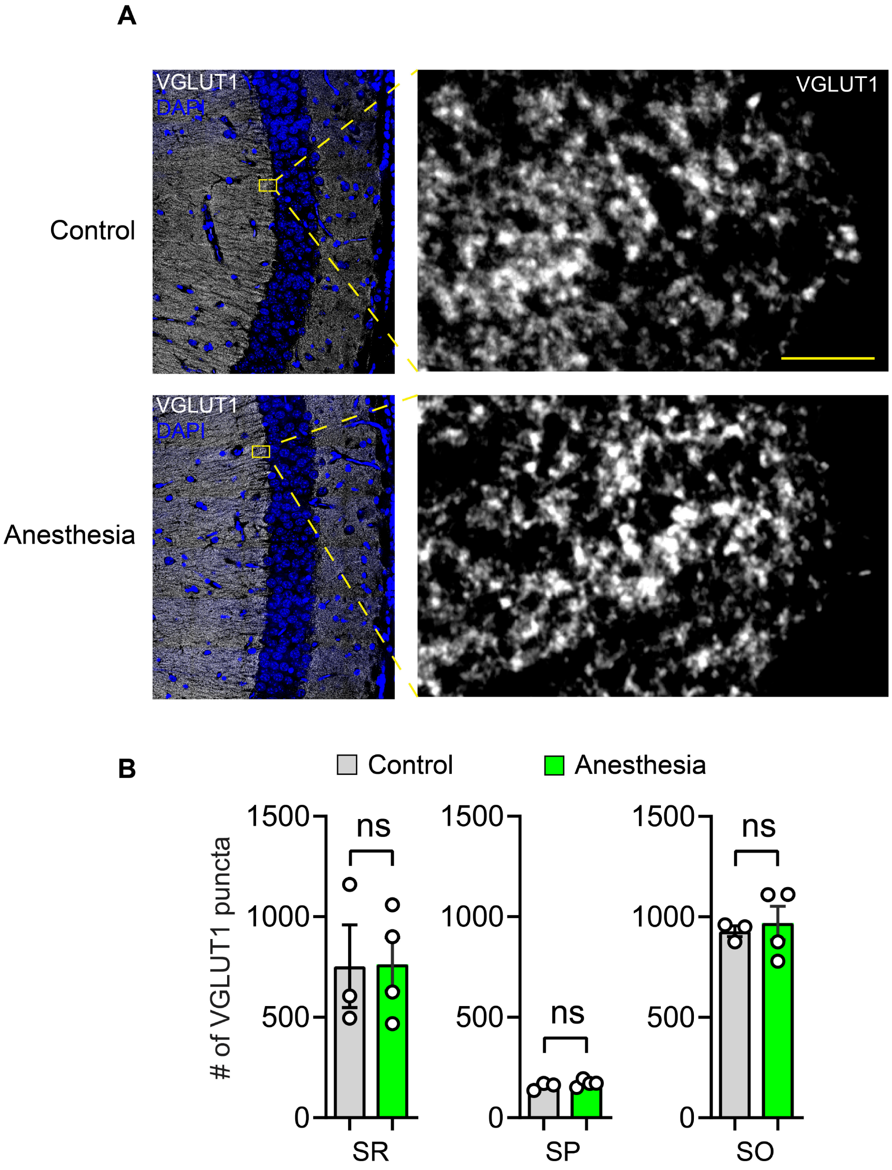

Supplemental Figure 3. The number of excitatory synapses does not change after repeated anesthesia.

No difference in the number of excitatory synapses after repeated anesthesia (PD 15-17) was observed (**A**) as the number of VGLUT1 puncta was not altered in the CA1 at PD 24 (**B**). SO – *stratum oriens*, SP – *stratum pyramidale*, SR – *stratum radiatum* (Control = 3 mice, Anesthesia = 4 mice, $p > 0.05$, unpaired 2-tailed *t*-test). Each data point represents individual male animal. All data are presented as mean ± s.e.m. Scale bar, 4 µm.

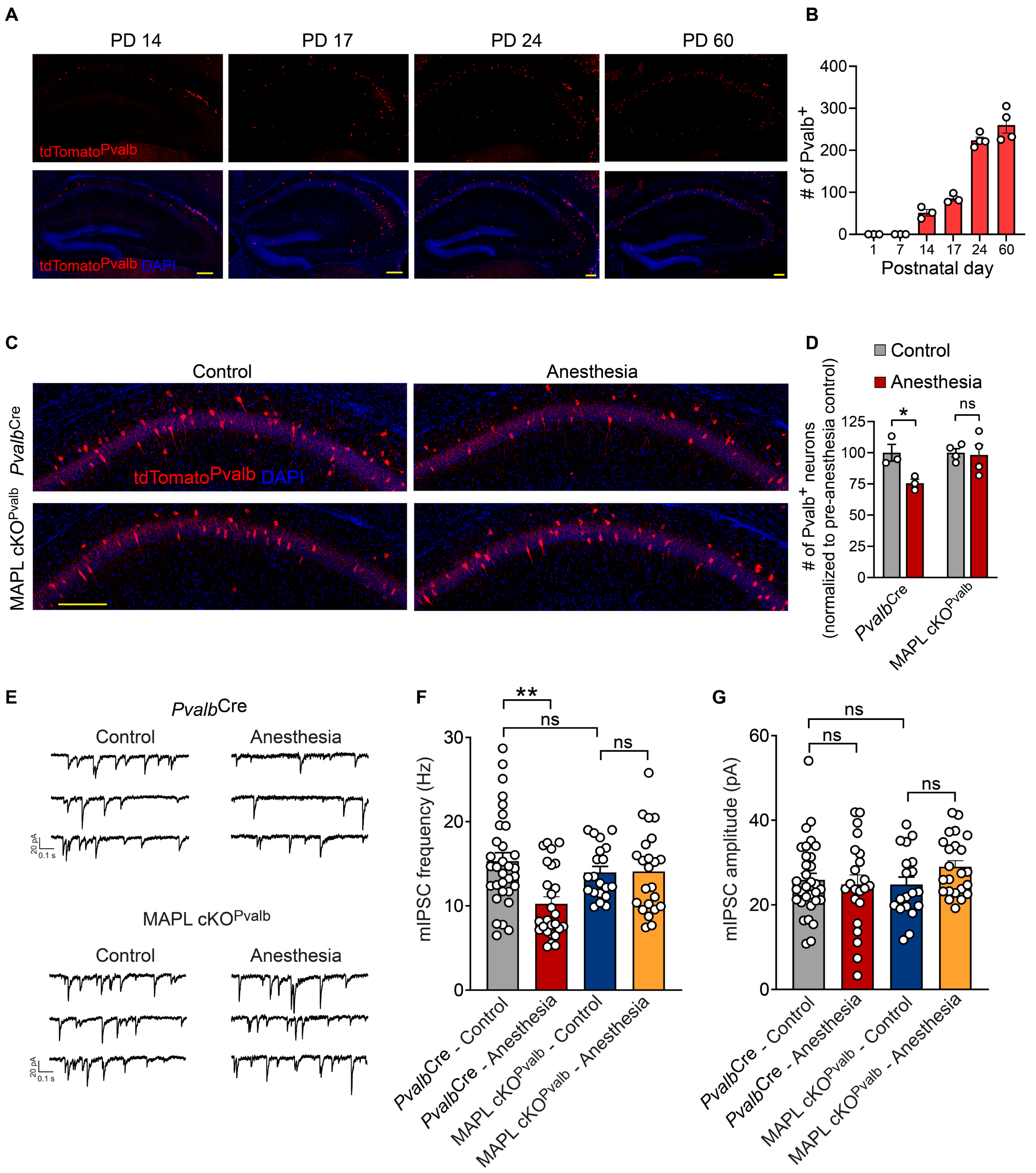

Supplemental Figure 4. Selective blocking of apoptosis in Pvalb neurons by ablation of MAPL prevents loss of Pvalb neurons and rescues reduced synaptic inhibition of hippocampal pyramidal neurons.

(**A**) The expression of Cre recombinase in tdTomato^{Pvalb} male mice was assessed by quantifying the number of hippocampal tdTomato^{+} cells in different postnatal time points (postnatal day 1, 7, 14, 17, 24, and 60). Scale bar, 200 μm. Each data point in **B** represents individual animal. (**C**) *Pvalb*^{Cre} and MAPL cKO^{Pvalb} male mice were subjected to repeated isoflurane anesthesia at PD 15-17 (2

hours a day) and brains were PFA-fixed 7 days after the last anesthesia session (PD 24) and processed for parvalbumin immunostaining. (**D**) Quantification shows that repeated anesthesia induces a reduction in Pvalb-positive cells in *Pvalb*^{Cre} mice but not in MAPL cKO^{Pvalb} animals (*Pvalb*^{Cre}: Control (n = 3 mice) versus Anesthesia (n = 3 mice), $t(10) = 2.691$, $p = 0.0448$; MAPL cKO^{Pvalb}: Control (n = 4 mice) versus Anesthesia (n = 4 mice), $t(10) = 0.2178$, $p = 0.9718$, statistics are based on two-way ANOVA followed by Tukey's post-hoc comparison). mIPSCs were recorded from CA1 pyramidal neurons at PD 24 from WT (*Pvalb*^{Cre}) and MAPL cKO^{Pvalb} male mice subjected to repeated anesthesia at PD 15-17. (**E**) Representative traces of recording in different groups. (**F**) mIPSC frequency was reduced in *Pvalb*^{Cre} mice subjected to anesthesia but not in MAPL cKO^{Pvalb} animals (*Pvalb*^{Cre} – Control (n = 32 cells from 10 mice) versus *Pvalb*^{Cre} – anesthesia (n = 26 cells from 8 mice), $q(31) = 5.57$, $p = 0.0023$; *Pvalb*^{Cre} – Control versus MAPL cKO^{Pvalb} – Control (n = 20 cells from 8 mice), $q(31) = 1.863$, $p = 0.559$; MAPL cKO^{Pvalb} – Control versus MAPL cKO^{Pvalb} Anesthesia (n = 23 cells from 9 mice), $q(31) = 0.588$, $p = 0.975$, statistics are based on nested one-way ANOVA followed by Tukey's post-hoc comparison, each data point in F-G represents individual cell). (**G**) No differences in mIPSC amplitude were found between groups ($p > 0.05$ for all comparisons, nested one-way ANOVA followed by Tukey's post-hoc comparison). All data are presented as mean ± s.e.m. *$p < 0.05$, **$p < 0.01$; ns, not significant.

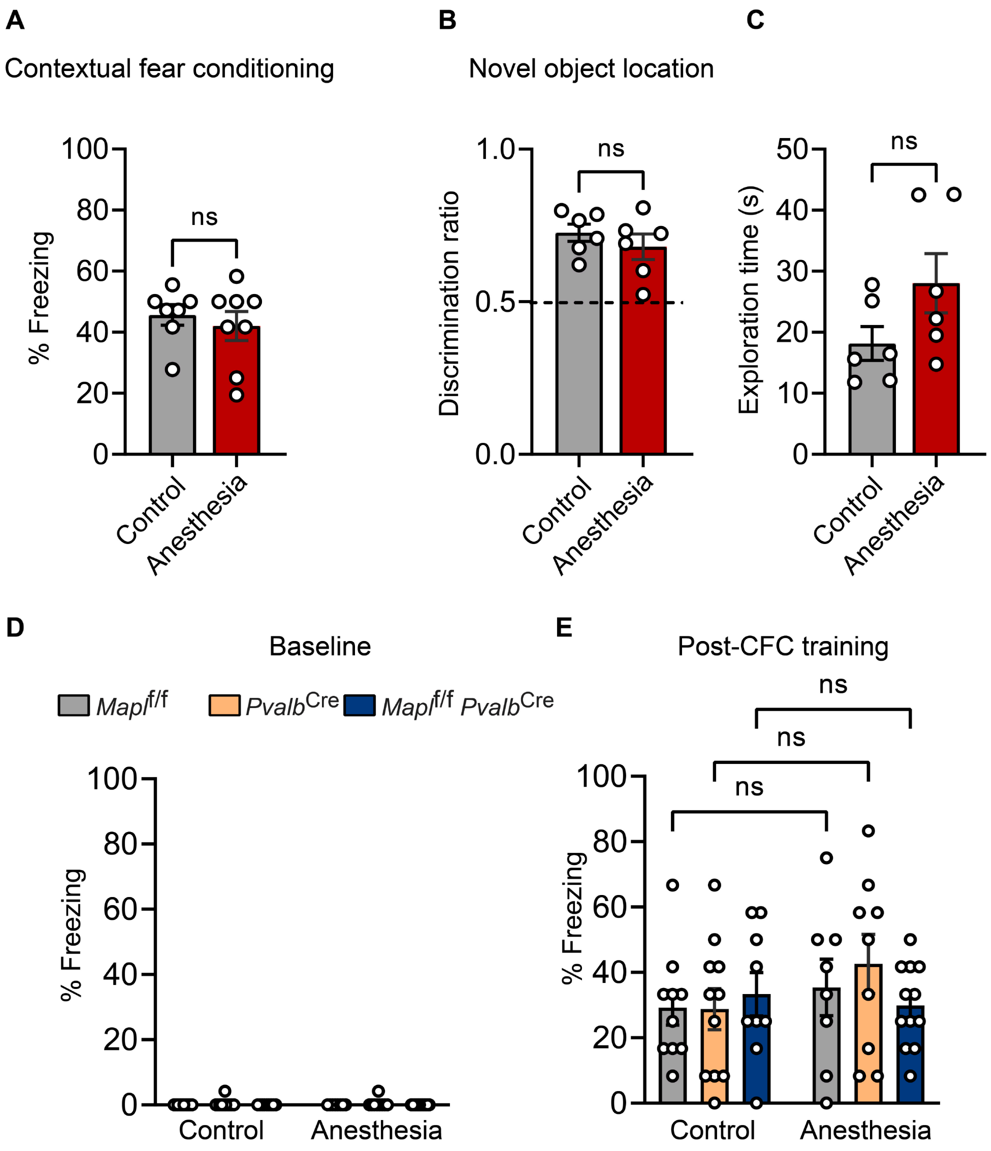

Supplemental Figure 5. Single two-hour-anesthesia exposure in juvenile mice does not impair long-term memory in adulthood.

Male mice were subjected to a single 2-hour session of isoflurane anesthesia at postnatal day 15 and the hippocampus-dependent memory was assessed in adulthood (PD 60) in contextual fear conditioning (**A**) and novel object location (**B**, **C**) tests. No difference in freezing behaviour between control and anesthesia-exposed mice was found 24 hours post-training in contextual fear conditioning (**A**, n = 7/8 mice, $t = 0.606$, $p = 0.55$, unpaired 2-tailed t-test). No memory deficits were found in anesthesia-exposed mice in object location test (**B**, n = 6/6 mice, $t = 0.91$, $p = 0.38$, unpaired 2-tailed t-test) and no differences were measured in total exploration (**C**, n = 6/6 mice, $t = 1.77$, $p = 0.106$, unpaired 2-tailed t-test). (**D**, **E**) Male mice were subjected to repeated general anesthesia at PD 15-17 (3 sessions of 2 hours per day) and subjected to contextual fear conditioning test at PD 60. Quantification of freezing behaviour before the foot shock (**D**, for 2 min) and immediately after the foot shock (**E**, for 1 min) showed no change in anesthesia-exposed mice ($p > 0.05$, one-way ANOVA followed by Tukey's post-hoc comparison). All data are presented as mean ± s.e.m. ns, not significant.

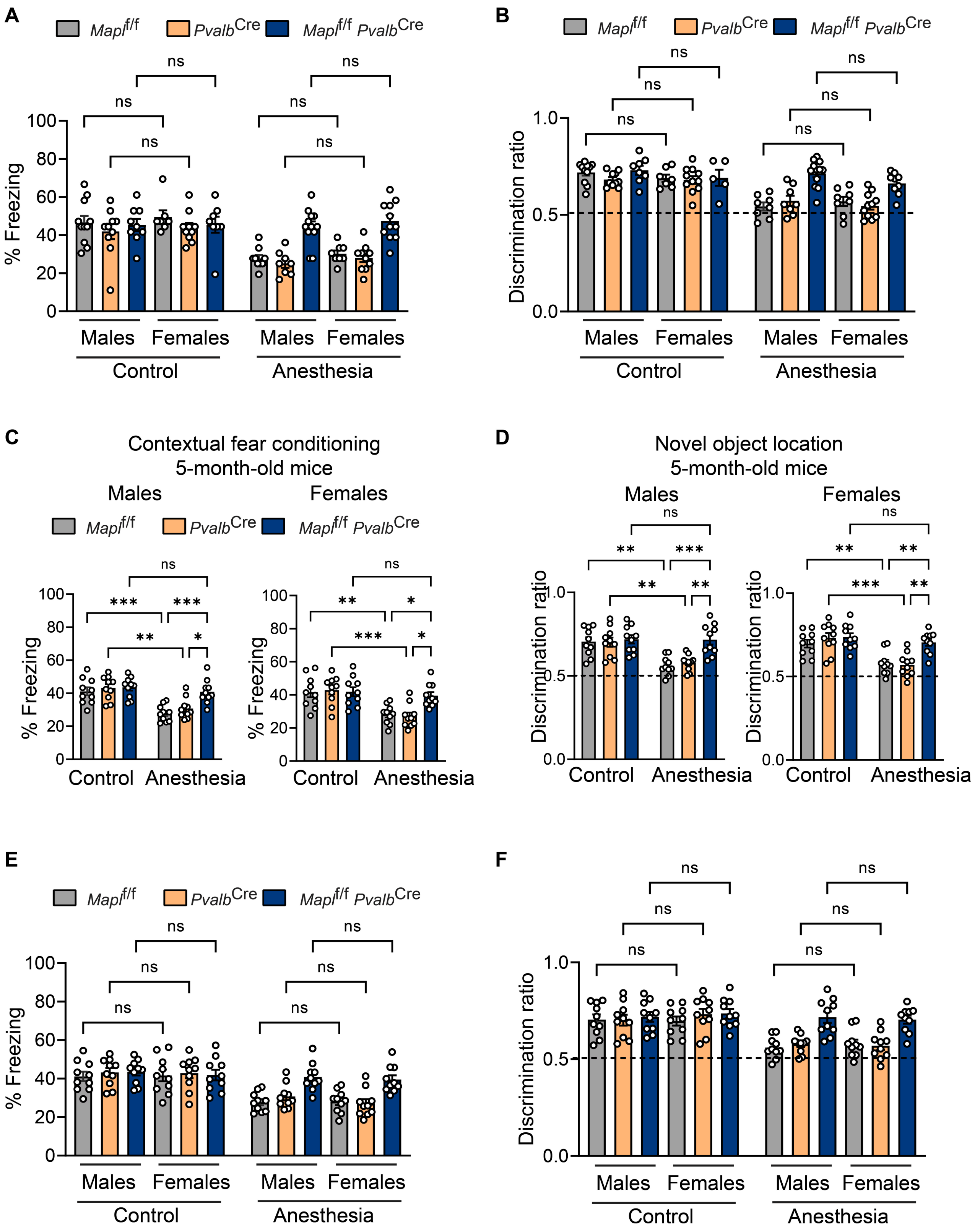

Supplemental Figure 6. No sex differences in anesthesia-induced memory deficits in 2-month-old mice and anesthesia-induced cognitive impairment in 5-month-old mice.

Data presented in Figure 4D-I were used to compare long-term memory in contextual fear conditioning (**A**) and novel object location (**B**) tasks between 2-month-old male and female control and anesthesia-exposed (PD 15-17) mice. No sex differences between males and females were found in either control or anesthesia-exposed groups (statistics are based on two-way ANOVA followed by Tukey's post-hoc comparison). New cohorts of male and female mice (10 animals/group) were subjected to repeated anesthesia at PD 15-17 and their long-term memory was assessed in contextual fear conditioning (24 post-training, **C**) and novel object location (**D**) tasks four and a half months later at the age of five months. Whereas MAPL$^{f/f}$ and Pvalb^{Cre} male and female mice exhibited memory deficits in both tasks, MAPL$^{f/f}$ Pvalb^{Cre} animals showed intact long-term memory. Comparison between males and females showed no sex differences in contextual fear conditioning (**E**) and novel object location (**F**) tasks. Statistics are based on two-way ANOVA followed by Tukey's post-hoc comparison All data are presented as mean ± s.e.m. *$p < 0.05$, **$p < 0.01$, ***$p < 0.001$; ns, not significant.

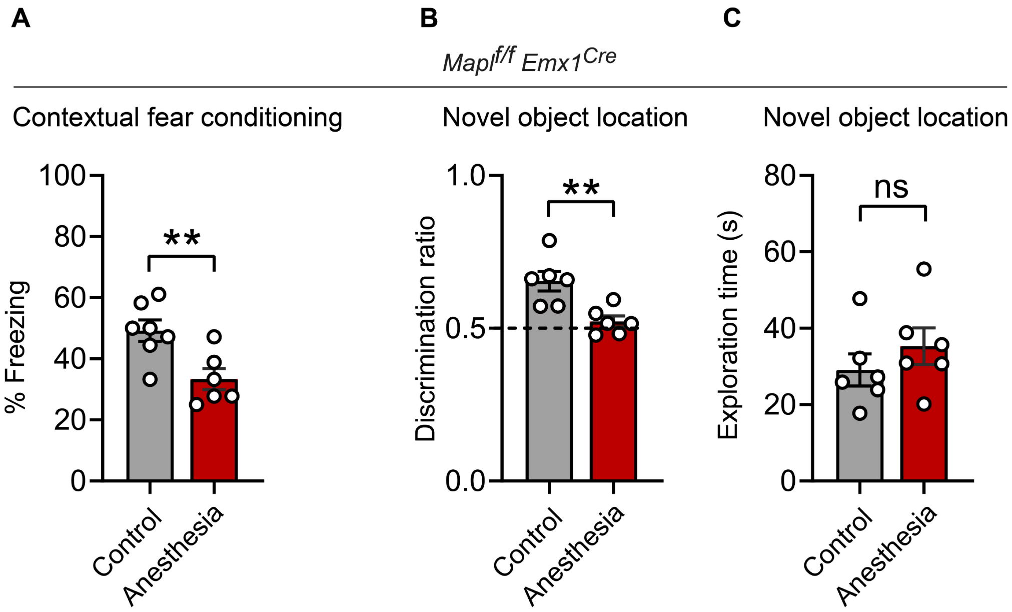

Supplemental Figure 7. Ablation of MAPL in excitatory neurons does not protect against repeated anesthesia-induced memory deficits in mature mice.
(**A**-**C**) *Mapl*$^{f/f}$ *Emx1*^{Cre} male mice, lacking MAPL in excitatory neurons, have been subjected to repeated isoflurane anesthesia at PD 15-17 and to behavioural testing at PD 60. *Mapl*$^{f/f}$ *Emx1*^{Cre} mice exhibited impaired long-term memory (24 post-training) in contextual fear conditioning (**A**, $t = 3.227$, $p = 0.0081$, unpaired 2-tailed *t*-test, n = 7/6) and novel object location (**B**, $t = 3.57$, $p = 0.0051$, unpaired 2-tailed *t*-test, n = 6/6) tests. No differences in exploration time were found between the groups in novel object location test (**C**, $p > 0.05$). All data are presented as mean ± s.e.m. $^{**}p < 0.01$, ns, not significant.
